# Genomic epidemiology of *Escherichia coli* isolates from a tertiary referral center in Lilongwe, Malawi

**DOI:** 10.1099/mgen.0.000490

**Published:** 2020-12-09

**Authors:** Gerald Tegha, Emily J. Ciccone, Robert Krysiak, James Kaphatika, Tarsizio Chikaonda, Isaac Ndhlovu, David van Duin, Irving Hoffman, Jonathan J. Juliano, Jeremy Wang

**Affiliations:** ^1^​ UNC Project, Lilongwe, Malawi; ^2^​ Division of Infectious Diseases, School of Medicine, University of North Carolina, USA; ^3^​ Kamuzu Central Hospital, Lilongwe, Malawi; ^4^​ Department of Epidemiology, Gillings School of Global Public Health, University of North Carolina, USA; ^5^​ Curriculum in Genetics and Molecular Biology, School of Medicine, University of North Carolina, USA; ^6^​ Department of Genetics, School of Medicine, University of North Carolina, USA

**Keywords:** *Escherichia coli*, molecular epidemiology, whole genome sequencing, antimicrobial resistance, Africa, Malawi

## Abstract

Antimicrobial resistance (AMR) is a global threat, including in sub-Saharan Africa. However, little is known about the genetics of resistant bacteria in the region. In Malawi, there is growing concern about increasing rates of antimicrobial resistance to most empirically used antimicrobials. The highly drug resistant *Escherichia coli* sequence type (ST) 131, which is associated with the extended spectrum β-lactamase *bla_CTX-M-15_*, has been increasing in prevalence globally. Previous data from isolates collected between 2006 and 2013 in southern Malawi have revealed the presence of ST131 and the *bla_CTX-M-15_* gene in the country. We performed whole genome sequencing (WGS) of 58 clinical *E. coli* isolates at Kamuzu Central Hospital, a tertiary care centre in central Malawi, collected from 2012 to 2018. We used Oxford Nanopore Technologies (ONT) sequencing, which was performed in Malawi. We show that ST131 is observed more often (14.9% increasing to 32.8%) and that the *bla_CTX-M-15_* gene is occurring at a higher frequency (21.3% increasing to 44.8%). Phylogenetics indicates that isolates are highly related between the central and southern geographic regions and confirms that ST131 isolates are contained in a single group. All AMR genes, including *bla_CTX-M-15_*, were widely distributed across sequence types. We also identified an increased number of ST410 isolates, which in this study tend to carry a plasmid-located copy of *bla_CTX-M-15_* gene at a higher frequency than *bla_CTX-M-15_* occurs in ST131. This study confirms the expanding nature of ST131 and the wide distribution of the *bla_CTX-M-15_* gene in Malawi. We also highlight the feasibility of conducting longitudinal genomic epidemiology studies of important bacteria with the sequencing done on site using a nanopore platform that requires minimal infrastructure.

## Data Summary

The sequencing data used for this analysis is available in public data repositories. Information on the newly generated data is available in NCBI SRA (BioProject ID PRJNA635644). Other publicly available sequences used are provided in Table S2.

**Impact Statement**

Antimicrobial resistance is a global public health emergency. Although rates of resistance are high in Africa, little is known about the genetics and resistance mechanisms of clinically important bacteria. Here we characterize the molecular epidemiology of *Escherichia coli* isolates from a tertiary referral hospital in Malawi and compare these with historical isolates from the same country. Consistent with their global expansion, we show that ST131 is observed more often and that the *bla_CTX-M-15_* gene is occurring at a higher frequency between studies. However, phylogenetics indicates that isolates are highly related between the central and southern geographic regions. This study highlights the feasibility of conducting longitudinal genomic epidemiology studies of important bacteria with the sequencing done on site using a nanopore platform that requires minimal infrastructure.

## Introduction

Antimicrobial resistance (AMR) is one of the most serious global public health threats [1]. Of specific concern are *Enterobacterales* (formerly the Enterobacteriaceae) that are resistant to third-generation cephalosporins such as ceftriaxone. The World Health Organization (WHO) has designated ceftriaxone-resistant *Enterobacterales* as a critical priority [2]. In sub-Saharan Africa (SSA), there is growing evidence that ceftriaxone-resistant *Enterobacterales* are important pathogens in invasive infections such as bacteremia. Given that ceftriaxone is often used to treat severe infections in SSA, and carbapenems are not often available, this is a major concern.

Among the *Enterobacterales *, *Escherichia coli* (* E. coli *) is a common cause of invasive disease, accounting for between 3 and 33 % of positive blood cultures in case series in Africa [3–8]. *E. coli* is becoming more resistant to commonly used antibiotics in SSA, including ceftriaxone. Additionally, recent evidence indicates that the highly drug resistant *E. coli* sequence type (ST) 131 has been increasing in prevalence globally [9–11]. This sequence type is an extraintestinal pathogenic *E. coli* (ExPEC) that is associated with bloodstream and urinary tract infections, often possessing genes associated with extended-spectrum β-lactamases (ESBLs) [12, 13]. The main mechanism of cephalosporin resistance is drug inactivation mediated by hydrolysis of the β-lactam ring by ESBL enzymes. Cefatoxamine-resistance Munich (CTX-M) derivatives are the dominant and most widely distributed ESBL enzymes among *E. coli* [14]. CTX-M-15 is strongly associated with ST131 [15–18]. The global spread of ESBL-* E. coli* is largely attributed to the dissemination of *E. coli* strains carrying the *bla_CTX-M-15_* gene, especially *E. coli* O25b:H4-ST131 [11]. Previously, three major lineages of ST131 have been identified that differed mainly with respect to their fimH alleles: A (mainly fimH41), B (mainly fimH22) and C (mainly fimH30) [19]. Clade C has predominated since the 2000s, corresponding with the rapid dissemination of the *bla_CTX-M-15_* allele [11, 19, 20]. There is growing evidence in SSA that ST 131 CTX-M-15 *E. coli* strains are increasing, but there remains a limited number of studies assessing the clonality of *E.coli*, the distribution of ST131 and the presence of *bla_CTX-M-15_* genes using whole genome sequencing [21–24].

In Malawi, specifically, there has been growing concern about increasing rates of antimicrobial resistance to most empirically used antimicrobials [25, 26]. A recent genomic epidemiology study of 94 *E. coli* isolates collected at Queen Elizabeth Central Hospital, a tertiary care centre in southern Malawi, and analysed by whole genome sequencing (WGS), has shown that ST131 is the most common ST in southern Malawi at 14.9% of isolates sequenced. CTX-M-15 was found in 21.4% of ST131 isolates, but occurred across 11 STs [27]. The purpose of our study is to increase our understanding of the genomic epidemiology of *E. coli* in Malawi by conducting WGS, using Oxford Nanopore Technologies (ONT) sequencing performed in Malawi, of isolates collected at a tertiary care hospital in central Malawi. We use this data to define the clonality, virulence genes and antimicrobial resistance genes in the central region of the country and to compare these results with those for southern Malawi.

## Methods

### Sample selection

Sixty isolates were selected from the UNC Project-Malawi archives for sequencing. Samples were collected between 2012 and 2018. Organisms were isolated using sheep blood agar (trypticase soy agar prepared with 5% defibrinated sheep blood) and MacConkey agar (selective and differential medium for Gram-negative rods) as primary culture media. Depending on the year of sample collection, identification was done either using conventional biochemical tests (TSI, indole, citrate and urease) and or Analytical Profile Index (API) 20E, a biochemical panel for identification and differentiation of members of the family Enterobacteriaceae. Isolates were selected for this study on the basis of diversity of phenotypic resistance pattern and clinical source of isolation, similarly to a previous study in Malawi [27]. One isolate was excluded from further analysis due to poor sequencing coverage, and one was excluded as it was identified as *Klebsiella *, resulting in 58 isolates included in all analyses.

### Antimicrobial resistance testing

The Kirby-Bauer disc diffusion method was used to measure the *in vitro* susceptibility of bacteria to antimicrobial agents at the UNC Project-Malawi microbiology laboratory at the time of isolate collection. Results were obtained with disc diffusion tests that use the principle of standardized methodology and zone diameter measurements correlated with minimum inhibitory concentrations (MICs) with strains known to be susceptible, intermediate, and resistant to various antibiotics. All aspects of the procedure were standardized as recommended by the Clinical and Laboratory Standards Institute (CLSI) in the document *‘Performance Standards for Antimicrobial Susceptibility Testing*’ [28].

### Whole genome sequencing

Overnight cultures of the isolates were grown in 5 ml of LB broth at 37 °C. Cell pellets from the broth culture were recovered from 1.5 ml centrifuged at 10 000 ***g*** for 2 min. Cell pellets were resuspended in 100 µl of nuclease-free water. DNA was extracted from the resuspended pellet using the Zymo Quick-DNA Microprep Kit (cell suspensions protocol) as per the manufacturer’s instructions (Zymo Research). The DNA was quantified using the dsDNA kit on a Qubit 2.0 (Thermo Fisher). Equal amounts of DNA (~100 ng) from each isolate were used for library preparation using the Rapid Barcoding (RBK-004) as per the manufacturer’s protocol (Oxford Nanopore Technologies). Pooled libraries of 12 isolates were run on a R9.4.1 flow cell on a MinION/MinIT for 24 h at UNC Project-Malawi. Each flow cell was washed once per protocol and a second set of 12 isolates were run for an additional 24 h or until all pores were exhausted.

### *De novo* genome assembly

Base calling of fast5 files was done with Guppy (version 3.4.5) with the R9.4.1 ‘high accuracy’ model [29]. Samples were demultiplexed and adapters/barcodes trimmed using a custom tool, depore (version 0.1; https://github.com/txje/depore). Reads for each isolate were *de novo* assembled using Flye (version 2.7) [30]. By Flye’s design and empirically [31], chimeric reads should not affect assembly performance and are much less prevalent in rapid (transposase-based) as opposed to ligation-based library preparation [32]. Assemblies were then polished four times with racon (version 1.3.2) and underwent a final polish with medaka (version 0.6.2) (http://github.com/nanoporetech/medaka) [33, 34].

### Typing of isolates

Each assembly was aligned using minimap2 to databases of known serotypes, fimH type, genomic and plasmid sequence types including plasmid incompatibility, virulence factors, and antimicrobial resistance genes, listed in Table S1 (available in the online version of this article) [35–40]. Matches were made for each assembly using similar cutoffs to those used by the Centre for Genomic Epidemiology (CGE; https://cge.cbs.dtu.dk/services/data.php) tools. Specifically, we required 60% of the feature to match at >90% sequence identity to serotype markers (fliC, wzx/wzy), fimH variant, virulence genes, antimicrobial resistance genes, and plasmid incompatibility group markers. For multi-locus sequence type (MLST) and fimH type, we report the closest hit (or multiple hits in case of a tie). Assembled contigs were identified as plasmids if they were found to contain at least one plasmid incompatibility type marker. For *bla_CTX-M-15_* analysis, contigs >600 kbp without plasmid markers are inferred to be genomic, in all but two cases they are ~5 Mbp. No plasmid markers were found on any contig >300 kbp.

### Species identification

We detected O and/or H serotypes and multi-locus sequence types in 59 of 60 presumed *E. coli* samples. The remaining sample appeared to have assembled well and a blast search revealed it was a strain of *Klebsiella *. We used this sequence as an outgroup in our phylogenetic analysis and otherwise excluded it from further analysis. An additional isolate (#31) was excluded from analysis for poor sequencing coverage, leaving 58 samples for all downstream analyses.

### Phylogenomics

**Fig. 1.**
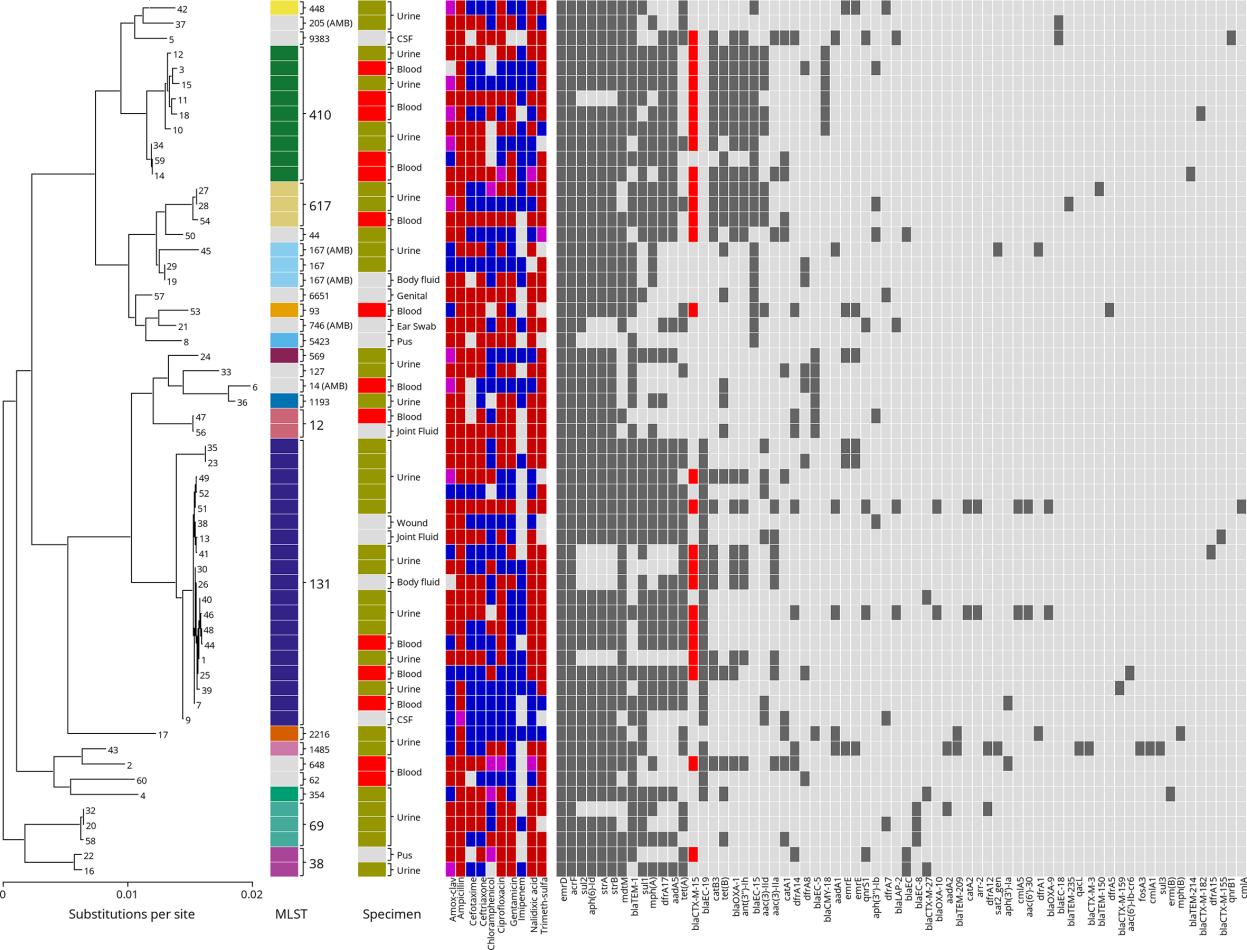
Distribution of sequence type, specimen type, resistance phenotype and resistance gene composition in 58 Malawian *E. coli* Isolates. Phylogenetic relationship among sequenced isolates with corresponding sequence type, specimen type, phenotypic and genomic AMR status. At the far left is the phylogeny relating these 58 samples with the sequenced *Klebsiella* from our study as an outgroup (not shown). In line with each terminal branch is the corresponding sample’s sequence type (ST) (‘AMB’ indicates ambiguity in ST assignment), specimen from which the sample was isolated, AMR phenotype (red: resistant, blue: susceptible, purple: intermediate, grey: unknown), and presence of each detected AMR gene in the genome assembly (dark grey: present, light grey: absent, red highlights presence of *bla_CTX-M-15_*).

**Fig. 2.**
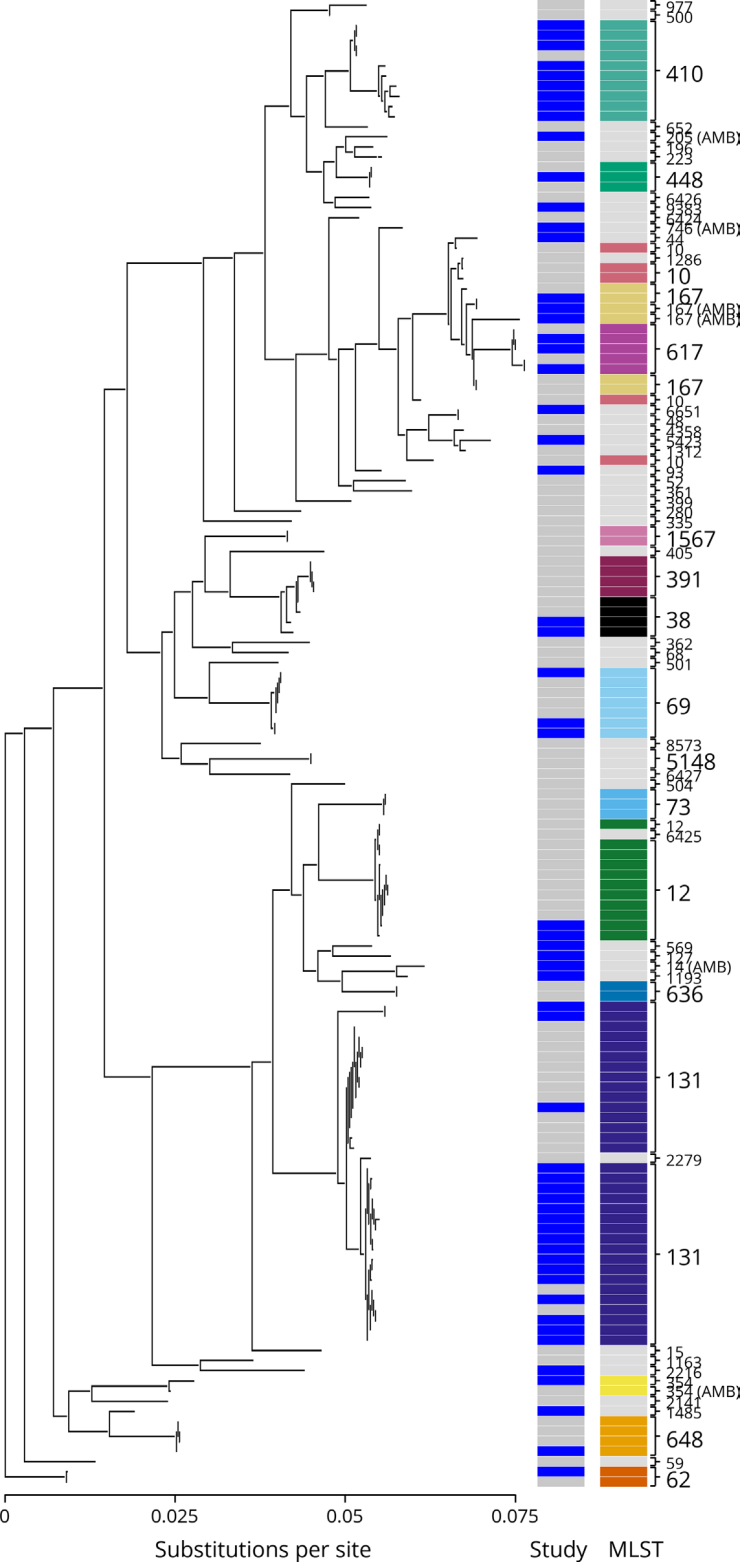
Phylogenetic tree of Malawian *E. coli* isolates. Phylogenetic relationships among the 58 isolates are presented here in relation to previously published Malawi *E. coli* isolates (Table S2). Samples cluster by sequence type, but are well mixed between the two geographically and temporally separated studies. In the left column, blue indicates isolates from this study and grey from the previous study [17]. The rightmost column indicates the MLST where sequence types occurring more than once are assigned a unique colour (all others are left grey).

Assembled genomes were aligned to a set of single-copy orthologs largely conserved across *Enterobacterales* (BUSCO v4, https://busco.ezlab.org/). A subset of 92 genes were selected that appear in all 58 samples that were included in the final analysis. A multiple-sequence alignment was performed for each gene using muscle (3.8.31) [41]. This concatenated alignment includes 108 278 sites, 16 729 informative SNPs. RAxML-ng was used to reconstruct a maximum-likelihood phylogenetic tree for the concatenated alignments with model parameter ‘GTR+G’ (Fig. 1) [42]. For the comparison to existing Malawi *E. coli* isolates, we used publicly available sequence data listed in Table S2. For each of these samples, a genome was assembled from Illumina sequence data with SPAdes (3.14.0) using default parameters [43]. A phylogeny incorporating these sequences was generated as described above, using a subset of 80 genes present in all 149 genomes (Fig. 2). This 92 gene alignment includes 125 994 sites and 18 282 informative SNPs. To evaluate the accuracy of these phylogenies based on a limited set of highly conserved genes in representing the global genomic phylogeny, we generated a more liberal ‘core’ gene alignment using Roary [44] that captures a much larger portion of the genome and found few differences (Supplemental Methods, Figures S1 and S2).

### Data analysis

Data were analysed using STATA/SE version 16.1 (StataCorp LLC, College Station, TX, USA). Descriptive statistics were used to describe clinical characteristics, frequencies of gene detections, and disc diffusion results. Given the small overall sample size, two-sided Fisher’s exact tests were used to assess for associations between genes detected and phenotypic resistance testing by Kirby–Bauer disc diffusion. *P*-values were corrected for multiple comparisons using the Benjamini–Hochberg false discovery rate method [45].

## Results

### Isolate characteristics and sequencing data

Five sequencing runs generated 9 291492 reads totalling 40.8 Gbp with a mean read length of 4391 bp and N50 of 8732 bp. Sequencing summary statistics across samples are described in Table 1. Clinical and patient characteristics for the isolates are summarized in Table 2. The majority were from a urinary source (59%) and were from females (74%). A significant number of isolates were from sterile sites, including blood (24%), cerebrospinal fluid (3%), and joint (3%). Patient white blood cell counts and haemoglobin levels were available for 20 of the isolates; medians and interquartile ranges are included in Table 2.

**Table 1.** Sequence data characteristics of all sequenced isolates

Sequence data	Value*
Number of reads	126 886 (21 843–539 714)
Total bps	556 Mbp (84–2076 Mbp)
Median fragment size sequenced in sample	2505 bp (673–4560 bp)
N_50_ assembly	4.88 Mbp (0.59–5.37 Mbp)
Number of contigs	5 (1–43)
Chromosomal median coverage	106 (15–392)

*Median (Minimum, Maximum).

**Table 2.** Patient characteristics of the 58 included isolates

Variable	Number of isolates n (%)
**Sex**	
Female	43 (74)
Male	15 (26)
Age*	34 (20–47)
**Sample Type**	
Blood	14 (24)
Urine	34 (59)
CSF	2 (3)
Body Fluid	2 (3)
Joint	2 (3)
Other	4 (7)
**Lab Values†**	
White blood cell count (cells µl^−1^)	7.5 (3.8–9.1)
Haemoglobin (g dl^−1^)	9.8 (8.4–11.6)

*Median (IQR); data available for 54 patients.

†Median (IQR); data available for 20 patients.

Kirby–Bauer antimicrobial susceptibility testing results are summarized in Table 3. Notably, the majority of isolates were resistant to amoxicillin and trimethoprim–sulfamethoxazole (TMP–SMX) and 57 % (33/58) were resistant to ceftriaxone. These are commonly prescribed antibiotics in the outpatient (amoxicillin and TMP–SMX) and inpatient (ceftriaxone) settings in Malawi [46].

**Table 3.** Kirby–Bauer antimicrobial resistance patterns of included isolates*

Drug (Total Tested)	Susceptible, n (%)	Intermediate, n (%)†	Resistant, n (%)
Amox-Clav (56)	15 (27)	9 (16)	32 (57)
Amoxicillin (58)	3 (5)	1 (2)	54 (93)
Cefotaxime (49)	21 (43)	0 (0)	28 (57)
Ceftriaxone (58)	25 (43)	0 (0)	33 (57)
Chloramphenicol (48)	29 (60)	4 (8)	15 (31)
Ciprofloxacin (58)	21 (36)	2 (3)	35 (60)
Gentamicin (57)	30 (53)	0 (0)	27 (47)
Imipenem (31)	31 (100)	0 (0)	0 (0)
Nalidixic Acid (57)	17 (30)	2 (4)	38 (67)
TMP-SMX (50)	4 (8)	1 (2)	45 (90)

*Percentage may not equal 100% due to rounding.

†Intermediate susceptibility by Kirby–Bauer test is defined based on a breakpoint that includes isolates with ‘zone diameters within the intermediate range that approach usually attainable blood and tissue levels.’ Response rates may be lower than for susceptible isolates; it implies clinical efficacy in sites where drugs physiologically concentrate or when a higher-than-normal dosage of a drug is used.

### Sequence types, H and O groups

Twenty-one different ST groups were found among 53 isolates. Five isolates could not be assigned to a unique ST group (Table S3). ST131 was the most common type identified [19 of 58 isolates (32.8%)], followed by ST410 which was present in 9 of 58 isolates (15.5%). Other STs that occurred in more than one isolate included ST69 (5.2%), ST38 (3.4%), ST617 (5.2%) and ST12 (3.4%). Of the 19 ST131, 18 of them were fimH30 and therefore clade C. One isolate was fimH27 and classified as clade B0. The fimH30 was linked to O25 and O16, while the fimH27 strain was O18 but also ST131. The population contained 23 different O groups (Table S4), with three samples being unable to be called, and 17 different H group calls (Table S5). A complete data set of genotype calls and sequencing characteristics is available in Table S6.

### Population structure of *E. coli* in Malawi

A phylogenetic tree of the isolates with previously reported *E. col *i genomes from southern Malawi [27] is shown in Fig. 2. Notably, the ST131 isolates cluster into a single group and have relatively little genetic distance between them in the tree.

### Genetic determinants of antimicrobial resistance

Our analysis identified 69 unique AMR genes that are known to encode proteins associated with antimicrobial susceptibility across a range of compounds (Table 4). AMR genes occurred across a range of ST and phylogenetic groups (Fig. 1).

**Table 4.** Prevalence of AMR genes identified

Gene	Resistance Phenotype	Isolates, n (%)
**Aminoglycoside Resistance**		
*aac(3)-IIa*	Gentamicin	11 (19)
*aac(3)-IId*	Gentamicin	15 (26)
*aac(6′)−30*	Aminoglycoside	2 (3)
*aac(6')-Ib-cr6*	Amikacin, kanamycin, tobramycin, quinolone	1 (2)
*aadA1*	Streptomycin	6 (10)
*aadA2*	Streptomycin	2 (3)
*aadA5*	Streptomycin	34 (59)
*ant(3'')-Ih*	Streptomycin/spectinomycin	20 (34)
*aph(3'')-Ib*	Streptomycin	5 (9)
*aph(3')-Ia*	Kanamycin	2 (3)
*aph(6)-Id*	Streptomycin	50 (86)
*strA*	Streptomycin	50 (86)
*strB*	Streptomycin	50 (86)
**Multidrug Efflux Pumps**		
*acrF*	Multidrug efflux	58 (100)
*emrD*	Multidrug efflux	58 (100)
*emrE*	Multidrug efflux	6 (10)
*mdtM*	Multidrug efflux	46 (79)
**β-lactam Resistance**		
*blaCMY-18*	Cephalosporin	6 (10)
*blaCTX-M-15*	Cephalosporin	26 (45)
*blaCTX-M-155*	Cephalosporin	1 (2)
*blaCTX-M-159*	Cephalosporin	1 (2)
*blaCTX-M-182*	Cephalosporin	1 (2)
*blaCTX-M-27*	Cephalosporin	3 (5)
*blaCTX-M-3*	Cephalosporin	1 (2)
*blaEC-15*	Cephalosporin	20 (34)
*blaEC-18*	Cephalosporin	2 (3)
*blaEC-19*	Cephalosporin	23 (40)
*blaEC-5*	Cephalosporin	7 (12)
*blaEC-8*	Cephalosporin	3 (5)
*blaEC*	β-lactam	3 (5)
*blaLAP-2*	β-lactam	4 (7)
*blaOXA-1*	Cephalosporin	21 (36)
*blaOXA-10*	Cephalosporin	2 (3)
*blaOXA-9*	β-lactam	2 (3)
*blaTEM-1*	β-lactam	43 (74)
*blaTEM-113*	Cephalosporin	1 (2)
*blaTEM-150*	β-lactam	1 (2)
*blaTEM-209*	β-lactam	2 (3)
*blaTEM-214*	β-lactam	1 (2)
*blaTEM-235*	β-lactam	1 (2)
**Chloramphenicol Resistance**		
*catA1*	Chloramphenicol	11 (19)
*catA2*	Chloramphenicol	2 (3)
*catB3*	Chloramphenicol	21 (36)
*cmlA*	Chloramphenicol	1 (2)
*cmlA1*	Chloramphenicol	1 (2)
*cmlA5*	Chloramphenicol	2 (3)
**TMP/SMX Resistance**		
*dfrA1*	Trimethoprim	2 (3)
*dfrA12*	Trimethoprim	2 (3)
*dfrA14*	Trimethoprim	9 (16)
*dfrA15*	Trimethoprim	1 (2)
*dfrA17*	Trimethoprim	35 (60)
*dfrA5*	Trimethoprim	1 (2)
*dfrA7*	Trimethoprim	4 (7)
*dfrA8*	Trimethoprim	8 (14)
*sul1*	Sulfonamide	39 (67)
*sul2*	Sulfonamide	51 (88)
*sul3*	Sulfonamide	1 (2)
**Quinolone Resistance**		
*qnrB1*	Quinolone	1 (2)
*qnrS1*	Quinolone	5 (9)
**Other Antimicrobials**		
*arr-2*	Rifamycin	2 (3)
*aar-3*	Rifamycin	1 (2)
*erm(B*)	Macrolide	1 (2)
*fosA3*	Fosfomycin	1 (2)
*mph(A*)	Macrolide	37 (64)
*mph(B*)	Macrolide	1 (2)
*qacL*	Quaternary Ammonium	1 (2)
*sat2_gen*	Streptothricin	2 (3)
*tet(A*)	Tetracycline	30 (52)
*tet(B*)	Tetracycline	21 (36)

### β-Lactam and ESBL resistance

We identified 23 genes associated with β-lactam resistance, including 10 ESBL genes (Table 4). Extended-spectrum β-lactamases included *bla_TEM-113_*, *bla_CTX-M-15_*, *bla_CTX-M-155_*, *bla_CTX-M-159_*, *bla_CTX-M-182_*, *bla_CTX-M-27_*, *bla_CTX-M-3_*, *bla_EC-15_*, *bla_EC-18_* and *bla_EC-19_*. Forty-eight out of 58 (83%) isolates carried at least one ESBL gene, and a total of 40 (69%) isolates carried more than one ESBL gene. The presence of any ESBL gene was associated with phenotypic resistance to ceftriaxone (*P*=0.004). Fifteen isolates with an ESBL gene retained phenotypic susceptibility to ceftriaxone by disc diffusion. All of these isolates each had only one of the following ESBL genes: *bla_EC-15_*, *bla_EC-18_*, *bla_EC-19_*.

The most common ESBL gene detected was *bla_CTX-M-15_*, which occurred in 26 (44.8%) isolates (Table 5). Presence of the *bla_CTX-M-15_* gene was associated with ceftriaxone resistance, with all 26 isolates being resistant to ceftriaxone by disc diffusion. The *bla_CTX-M-15_* gene was found in isolates of eight different sequence types, most commonly ST131 [10 out of 19 isolates (52.6%)] and ST410 [8 out of 9 (88.9%) isolates]. We identified 15 O25b:H4-ST131 isolates, of which 10 (66%) had the *bla_CTX-M-15_* gene. In ST131, the *bla_CTX-M-15_* gene was highly associated with a chromosomal location with eight isolates (80%) having chromosomal copies, one isolate had a plasmid copy and one isolate with the gene in both locations. ST410 had a very different distribution with six of the eight isolates (75%) containing the *bla_CTX-M-15_* gene in a plasmid-mediated copy, while only a single isolate had a chromosomal copy and a single isolate had it in both locations.

**Table 5.** Characteristics of CTX-M-15 associated isolates and ceftriaxone disk diffusion results

Isolate	Year Collected	Source	Sequence Type (ST)	Plasmid Inclusion Group(s)	Disk Diffusion Result	Location of Gene
1	2018	Urine	131	incB/O/K/Z	R	Genomic
2	2017	Blood	648	incF	R	Genomic
3	2018	Urine	410	incF	R	Plasmid
5	2018	Blood	9398	incHI2, incY	R	Plasmid
10	2018	Body Fluid	410	incF	R	Plasmid
11	2017	Urine	410	incF	R	Plasmid
12	2017	Urine	410	incF	R	Both
14	2014	Pus	410	incF, incY	R	Plasmid
15	2018	Urine	410	incF	R	Plasmid
18	2018	Body Fluid	410	incF	R	Genomic
22	2018	Blood	38	incF	R	Plasmid
25	2014	Urine	131	incF	R	Genomic
26	2014	Urine	131	incF	R	Plasmid
27	2015	Urine	617	incF	R	Both
28	2014	Urine	617	incF, incN	R	Plasmid
30	2016	Urine	131	incF	R	Genomic
34	2016	Urine	410	incF	R	Plasmid
41	2013	Blood	131	incN	R	Genomic
44	2013	Urine	131	incF	R	Genomic
46	2013	Urine	131	incF	R	Genomic
48	2014	Blood	131	incF	R	Genomic
49	2013	Blood	131	incF	R	Genomic
50	2013	Joint Fluid	44	incF	R	Plasmid
51	2013	Genital Swab	131	incF	R	Both
53	2015	Blood	93	incI-1I, incF	R	Plasmid
54	2013	Cerebrospinal Fluid	617	incF	R	Plasmid

### Fluoroquinolone resistance

We extracted the sequence of the *gyrA* gene from each isolate and translated them into protein sequences. We examined these for the presence of *gyrA* mutations that have previously been described in Malawi [27]. We identified the S83L mutation in 24 isolates and the D87N mutation in 22 isolates. In all cases, the D87N mutation co-occurs with the S83L mutation; in only two cases was a single mutation (S83L) observed. In addition, we identified the *qnrB1* gene and the *qnrS1* gene in 1 (2%) and 5 (9%) of isolates, respectively. These genes did not co-occur in isolates with *gyr* mutations.

### Aminoglycoside resistance

We identified 13 genes associated with aminoglycoside resistance in these isolates (Table 4). Several genes were associated with gentamicin resistance by disc diffusion, *aac(3)-IIa* (*P*<0.001), *aac(3)-IId* (*P*<0.001), and *ant(3″)-Ih* (*P*=0.004). We also detected *strA* or *strB* in combination in 50 of the isolates, as has been seen in southern Malawi previously [27]. When found together, these genes confer resistance to streptomycin, which is used in tuberculosis therapy in Africa [47].

### Resistance to other antimicrobials

We identified six genes associated with chloramphenicol resistance, the most common being *catA1* in 11 (19%) of isolates and *catB3* in 21 (36%) of isolates. We did not identify isolates with *floR*, which has previously been identified in southern Malawi [27]. Detection of *catA1* was associated with intermediate susceptibility or resistance to chloramphenicol by disc diffusion (*P*=0.004), but detection of *catB3* was not (*P*=0.613). We identified 11 genes associated with resistance to TMP-SMX, the most common being the trimethoprim resistance gene *dfrA17* (60% of isolates) and the sulfonamide resistance genes *sul1* (67% of isolates) and *sul2* (88% of isolates). Of these three genes, only *sul2* detection was associated with TMP-SMX intermediate susceptibility or resistance by disc diffusion (*P*=0.025); all isolates that were phenotypically intermediate or resistant to TMP-SMX had *sul1* and/or *sul2*. We detected *sul1* and *sul2* in 35 of the 58 isolates. Finally, we identified a handful of resistance genes to other antimicrobials, including rifamycin, macrolides, fosfomycin, and tetracyclines (Table 4). Interestingly, the most common AMR genes detected were multidrug efflux pumps, *acrF* and *emrD*, both of which occurred in all isolates.

### Plasmid incompatibility group

We identified eight different plasmid incompatibility groups (Table 6). The most common were incF incompatibility groups, at least one variant of which (incFIA/incFIB/incFII) was found in all but seven isolates (88%). Although the majority of ST131 were in this group, it was not associated with ST131 (*P*=0.567) as many non-ST131 isolates had incF incompatibility groups as well. Please see Table S6 for all inc type combinations.

**Table 6.** Prevalence of plasmid incompatibility groups

Plasmid Incompatibility Group	Number of isolates, n (%)
incFII	26 (45)
incFIA	19 (33)
incFIB	15 (26)
incY	4 (7)
incB/O/K/Z	2 (3)
incI	2 (3)
incI1-I	2 (3)
incN	2 (3)

### Virulence genes

In total, we identified 37 virulence genes (Table 7) with each isolate carrying a median of seven genes (IQR 3–10). The virulence factors spanned a range of functions, including acid resistance, adhesion, invasins, metalloproteases, and toxins. The most common gene detected was *gad,* which occurred in all 58 isolates and encodes glutamate decarboxylase, an enzyme linked with bacterial ability to resist environmental stresses [48]. Multiple adhesin proteins were also identified. The most common of these were *papC*, *papH*, *papG-II*, and *IpfA. papC* (36% of isolates), *papH* (35% of isolates), *papG-II* (35% of isolates) are all involved in pili function. *IpfA* encodes the long polar fimbriae associated with human diarrheal disease, and occurred in 19 of 58 isolates [49]. A single protectin encoded by *iss* was identified and was the second most common virulence factor identified in 35 of 58 isolates. The *iss* (increased serum survival) gene was first identified in a human septicemic *E. coli* isolate and was associated with a 20-fold increase in complement resistance and a 100-fold increase in virulence toward 1-day-old chicks [50–52]. Multiple siderophores were identified, with the most common being *iha*, occurring in 29 of 58 isolates [53]. We also identified two common toxins, *sat* which occurred in 23 of 58 isolates and *senB* in 22 of 58 isolates. The secreted autotransporter toxin (*sat*) appears to fall within one subgroup of autotransporters recently classified as the serine protease autotransporters of *Enterobacteriaceae* (SPATE) family. It acts as a vacuolating cytotoxin for bladder and kidney epithelial cells [54]. The *senB* gene encodes the TieB protein, which may have some role in enterotoxicity of EIEC [55, 56].

**Table 7.** Prevalence of virulence genes identified

Category	Gene	Isolate, n (%)
**Acid resistance**	gad*	58 (100)
**Adhesin**	*afaC*	13 (22)
	*air*	9 (16)
	*lpfA*	19 (33)
	*nfaE*	13 (22)
	*papA*	1 (2)
	*papC*	21 (36)
	*papE*	2 (3)
	*papG-II*	20 (35)
	*papG-III*	1 (2)
	*papH*	20 (35)
	*sfaF*	3 (5)
	*sfaS*	1 (2)
**Effector (T3SS**)	*espl*	1 (2)
**Invasin**	*ibeA*	1 (2)
**Metalloprotease**	*sslE*	23 (40)
**Microcin**	*mchB*	2 (3)
	*mchC*	3 (5)
	*mchF*	6 (10)
	*mcmA*	3 (5)
**Protectin**	*iss*	35 (60)
**Regulator**	*eilA*	9 (16)
**Siderophore**	*iha*	29 (50)
	*ireA*	6 (10)
	*iroN*	8 (13)
	*iutA*	3 (5)
**Toxin**	*cma*	1 (2)
	*cnf1*	6 (10)
	*hly-alpha*	9 (16)
	*pic*	1 (2)
	*sat*	23 (40)
	*senB*	22 (38)
	*tsh*	1 (2)
	*vat*	6 (10)
**Transferase**	*capU*	7 (12)
**ATP-binding cassette transporter**	*ybtP*	20 (35)
	*ybtQ*	22 (38)

*This gene is almost universal to *E.coli and* can be found in non-pathogenic strains.

## Discussion

In this study, we sequenced 58 *E. coli* isolates collected at a tertiary care centre in Lilongwe, Malawi between 2012–2018. To our knowledge, this is one of the first studies from Malawi demonstrating the feasibility of performing all steps of the whole genome sequencing process, including DNA extraction, library preparation, and the sequencing itself, on site in a local laboratory.

Relatively little data exist concerning the genomics of *E. coli* in Africa to date [21–24]. The isolates sequenced as part of our project collected in Lilongwe, in the central region of Malawi, are highly phylogenetically similar to those seen in a previous study conducted in Blantyre, Malawi, in the southern region [27]. Similarly to the previous report, we identified a diverse set of AMR genes, with similar genes occurring across a range of *E. coli* lineages in Malawi. We also provide additional information on common virulence genes associated with *E. coli* in Malawi, many of which are shared across a range of lineages.

In this joint phylogenetic analysis, we combined nanopore-only assembled genomes with solely short-read-based assemblies. This approach has the potential to introduce biases in both gene prediction and single-nucleotide polymorphisms due to the divergent error profiles of the two technologies. Nanopore sequencing in particular is known to suffer from a relatively high error rate among raw reads that is dominated by short insertions and deletions and primarily in homopolymer stretches [57]. Although we sequenced to sufficient depth that the vast majority of these errors were eliminated during the consensus generation and multiple polishing steps that produced the final assembly, undoubtedly there existed some residual errors above the rate typically observed with short-read sequencing platforms like Illumina [58]. Where these platform-specific errors are shared among samples sequenced using the same platform, there is the possibility of these segregating biases making their way into the predicted phylogeny. While we observe appropriate clustering of clades by sequence type regardless of sequencing approach (Fig. 2), there is, in several cases, apparent segregation of samples within STs (notably ST131) that is consistent with either true phylogenetic structure or segregating sequencing platform-specific biases. Since the samples collected in our study and the previous study [27] are separated both temporally and geographically, it is reasonable to expect within-ST variation to be attributable to segregating biological variation, but we cannot conclusively rule out that sequencing bias contributes to this phenomenon without careful manual inspection of the segregating sites.

Consistent with the Blantyre study’s findings [27], we show that *E. coli* is highly diverse, with a distribution of STs similar to global isolates. Importantly, the previous study utilized samples collected between 1996 and 2014, allowing temporal comparisons between studies as the majority of samples in our analysis were collected after 2013. Our results indicate that ST 131 has become more prevalent (14.9% increasing to 32.8%) and that the *bla_CTX-M-15_* gene is occurring at a higher frequency (21.3% increasing to 44.8%) in the intervening years. This is consistent with global trends that indicate that the highly drug-resistant *E. coli* ST131, associated with the *bla_CTX-M-15_* gene, has been increasing in prevalence [9–11, 15–18] As expected, there is some subtle structure within ST131, with isolates from this study being primarily localized on a branch with a longer internal branch length (Fig. 2). Another difference from previous work is the higher number of ST410 isolates in this study, which carried the *bla_CTX-M-15_* gene at the highest frequency of all the sequence types. Overall, there does not appear to be any strong associations between overall pattern of AMR gene content, virulence gene content, isolation site, and ST within the isolates (Fig. 1), similarly to previous results [27].

Although globally there is a strong association between ST131 and presence of the *bla_CTX-M-15_* gene, here we identified the *bla_CTX-M-15_* gene across a diverse set of lineages [11]. This finding is similar to those from other studies in Tanzania and Malawi, where the *bla_CTX-M-15_* gene was found across numerous STs [22, 27]. We see the *bla_CTX-M-15_* gene in eight different sequence types, including ST131. The ST that most commonly contained the *bla_CTX-M-15_* gene was ST410. All of the ST131 isolates that contained the *bla_CTX-M-15_* gene were O25b:H4-ST131 in this study. Overwhelmingly, the ST131 isolates were clade C, containing the fimH30 gene, consistent with the global expansion of ST131-H30 [12]. Interestingly, there is a strong association between ST and where the *bla_CTX-M-15_* gene is carried in the ST131 and ST410 isolates. ST131 was much more likely to have a genomic location for the gene, whereas ST410 more frequently carried the gene on a plasmid. This may have implications for how the *bla_CTX-M-15_* gene spreads in Malawi. Given the diverse lineages that carry the *bla_CTX-M-15_* gene, additional studies are needed to better understand the epidemiology of this gene in Malawi.

Overall, the patterns of AMR gene prevalence were similar to the one previous report from southern Malawi. *sul2*, *strA*, *strB*, *dfrA*, *bla_TEM-1_*, and *sul1* genes remained very common in the population. Interestingly, chloramphenicol resistance gene prevalence was lower than previously reported, with a decrease in the prevalence of *catA* from 64.9 % to 22 % [27]. This potentially reflects decreased use of the drug in the community with changing treatment guidelines and increasing availability of alternative agents with fewer side effects [46]. Most resistance genes were detected broadly across different genotypes in this study (Fig. 1).

In summary, we confirm that the *E. coli* population in Malawi is highly diverse, with evidence for increasing prevalence of the ST131 group in the country. We see a higher proportion of ST 131 isolates and a higher prevalence of the *bla_CTX-M-15_* gene in our isolates, which were collected a few years later than those described in previous reports. This expansion is consistent with the global increase in O25b:H4-ST131 bearing the fimH30 gene. *E. coli* genotypes are similar between two major tertiary care hospitals that are quite distant, with highly related isolates being found between the sites. As previously seen, AMR genes, including the *bla_CTX-M-15_* gene, are broadly contained across sequence types. A high diversity of virulence genes were seen within *E. coli* isolates. These data were collected by conducting ONT sequencing in Malawi, highlighting the possibility of conducting rapid longitudinal genomic epidemiology studies of consequential bacteria in sub-Saharan Africa where the sequencing is conducted on site.

## Supplementary Data

Supplementary material 1

Supplementary material 2

## References

[1] Prestinaci F, Pezzotti P, Pantosti A (2015). Antimicrobial resistance: a global multifaceted phenomenon. Pathog Glob Health.

[2] WHO WHO publishes list of bacteria for which new antibiotics are urgently needed. http://www.who.int/mediacentre/news/releases/2017/bacteria-antibiotics-needed/en/ (accessed 3 March 2020). http://www.who.int/mediacentre/news/releases/2017/bacteria-antibiotics-needed/en/.

[3] Hill PC, Onyeama CO, Ikumapayi UNA, Secka O, Ameyaw S (2007). Bacteraemia in patients admitted to an urban hospital in West Africa. BMC Infect Dis.

[4] Okomo UA, Garba D, Fombah AE, Secka O, Ikumapayi UNA (2011). Bacterial isolates and antibiotic sensitivity among Gambian children with severe acute malnutrition. Int J Pediatr.

[5] Nielsen MV, Sarpong N, Krumkamp R, Dekker D, Loag W (2012). Incidence and characteristics of bacteremia among children in rural Ghana. PLoS One.

[6] Enweronu-Laryea CC, Newman MJ (2007). Changing pattern of bacterial isolates and antimicrobial susceptibility in neonatal infections in Korle BU teaching Hospital, Ghana. East Afr Med J.

[7] Samuel SO, Fadeyi A, Akanbi AA, Ameen NB, Nwabuisi C (2006). Bacterial isolates of blood cultures in patients with suspected septicaemia in Ilorin, Nigeria. Afr J Med Med Sci.

[8] Popoola O, Kehinde A, Ogunleye V, Adewusi OJ, Toy T (2019). Bacteremia among febrile patients attending selected healthcare facilities in Ibadan, Nigeria. Clin Infect Dis.

[9] Poolman JT, Wacker M (2016). Extraintestinal pathogenic *Escherichia coli*, a common human pathogen: challenges for vaccine development and progress in the field. J Infect Dis.

[10] Decano AG, Downing T (2019). An *Escherichia coli* ST131 pangenome atlas reveals population structure and evolution across 4,071 isolates. Sci Rep.

[11] Ludden C, Decano AG, Jamrozy D, Pickard D, Morris D (2020). Genomic surveillance of *Escherichia coli* ST131 identifies local expansion and serial replacement of subclones. Microb Genom.

[12] Banerjee R, Johnson JR (2014). A new clone sweeps clean: the enigmatic emergence of *Escherichia coli* sequence type 131. Antimicrob Agents Chemother.

[13] Findlay J, Gould VC, North P, Bowker KE, Martin Williams O Characterisation of cefotaxime-resistant urinary *Escherichia coli* from primary care in south-west England.

[14] Chaudhuri RR, Henderson IR (2012). The evolution of the *Escherichia coli* phylogeny. Infect Genet Evol.

[15] D'Andrea MM, Arena F, Pallecchi L, Rossolini GM (2013). CTX-M-Type β-lactamases: a successful story of antibiotic resistance. Int J Med Microbiol.

[16] Peirano G, Richardson D, Nigrin J, McGeer A, Loo V (2010). High prevalence of ST131 isolates producing CTX-M-15 and CTX-M-14 among extended-spectrum-β-lactamase-producing *Escherichia coli* isolates from Canada. Antimicrob Agents Chemother.

[17] Peirano G, Costello M, Pitout JDD (2010). Molecular characteristics of extended-spectrum β-lactamase-producing *Escherichia coli* from the Chicago area: high prevalence of ST131 producing CTX-M-15 in community hospitals. Int J Antimicrob Agents.

[18] Kanamori H, Parobek CM, Juliano JJ, Johnson JR, Johnston BD (2017). Genomic analysis of multidrug-resistant *Escherichia coli* from North Carolina community hospitals: ongoing circulation of CTX-M-Producing ST131-*H*30Rx and ST131-*H*30R1 strains. Antimicrob Agents Chemother.

[19] Matsumura Y, Pitout JDD, Peirano G, DeVinney R, Noguchi T (2017). Rapid identification of different *Escherichia coli* sequence type 131 clades. Antimicrob Agents Chemother.

[20] Cantón R, González-Alba JM, Galán JC (2012). CTX-M enzymes: origin and diffusion. Front Microbiol.

[21] Irenge LM, Ambroise J, Bearzatto B, Durant J-F, Chirimwami RB (2019). Whole-genome sequences of multidrug-resistant *Escherichia coli* in South-Kivu Province, Democratic Republic of Congo: characterization of phylogenomic changes, virulence and resistance genes. BMC Infect Dis.

[22] Sonda T, Kumburu H, van Zwetselaar M, Alifrangis M, Mmbaga BT (2018). Whole genome sequencing reveals high clonal diversity of *Escherichia coli* isolated from patients in a tertiary care hospital in Moshi, Tanzania. Antimicrob Resist Infect Control.

[23] Ingle DJ, Levine MM, Kotloff KL, Holt KE, Robins-Browne RM (2018). Dynamics of antimicrobial resistance in intestinal *Escherichia coli* from children in community settings in South Asia and sub-Saharan Africa. Nat Microbiol.

[24] Mbelle NM, Feldman C, Osei Sekyere J, Maningi NE, Modipane L (2019). The resistome, mobilome, virulome and phylogenomics of multidrug-resistant *Escherichia coli* clinical isolates from Pretoria, South Africa. Sci Rep.

[25] Iroh Tam P-Y, Musicha P, Kawaza K, Cornick J, Denis B (2019). Emerging resistance to empiric antimicrobial regimens for pediatric bloodstream infections in Malawi (1998-2017). Clin Infect Dis.

[26] Haigh K, Dube Q, Kasambara W, Feasey NA, Lester R (2020). Cephalosporin resistance in Malawi. Lancet Infect Dis.

[27] Musicha P, Feasey NA, Cain AK, Kallonen T, Chaguza C (2017). Genomic landscape of extended-spectrum β-lactamase resistance in *Escherichia coli* from an urban African setting. J Antimicrob Chemother.

[28] Clinical and Laboratory Standards Institute M100 Performance Standards for Antimicrobial Susceptibility Testing. https://clsi.org/media/2663/m100ed29_sample.pdf (accessed 9 May 2020). https://clsi.org/media/2663/m100ed29_sample.pdf.

[29] nanoporetech nanoporetech/flappie. *GitHub*. https://github.com/nanoporetech/flappie (accessed 3 March 2020). https://github.com/nanoporetech/flappie.

[30] Kolmogorov M, Yuan J, Lin Y, Pevzner PA (2019). Assembly of long, error-prone reads using repeat graphs. Nat Biotechnol.

[31] Wick RR, Holt KE (2019). Benchmarking of long-read assemblers for prokaryote whole genome sequencing. F1000Res.

[32] Tvedte ES, Gasser M, Sparklin BC, Michalski J, Zhao X Comparison of long read sequencing technologies in resolving bacteria and fly genomes.

[33] Vaser R, Sović I, Nagarajan N, Šikić M (2017). Fast and accurate *de novo* genome assembly from long uncorrected reads. Genome Res.

[34] Website Medaka: sequence correction provided by ONT research. https://github.com/nanoporetech/medaka.

[35] Inouye M, Dashnow H, Raven L-A, Schultz MB, Pope BJ (2014). SRST2: rapid genomic surveillance for public health and hospital microbiology labs. Genome Med.

[36] Gonzalez-Escalona N, Allard MA, Brown EW, Sharma S, Hoffmann M Nanopore sequencing for fast determination of plasmids, phages, virulence markers, and antimicrobial resistance genes in Shiga toxin-producing *Escherichia coli*.

[37] Mohsin M, Raza S, Schaufler K, Roschanski N, Sarwar F (2017). High prevalence of CTX-M-15-Type ESBL-producing *E. coli* from migratory avian species in Pakistan. Front Microbiol.

[38] Shropshire WC, Aitken SL, Pifer R, Kim J, Bhatti MM Concurrence of porin loss and modular amplification of β-lactamase encoding genes drives carbapenem resistance in a cohort of recurrent Enterobacterales bacteremia. BioRxiv.

[39] Li H (2018). Minimap2: pairwise alignment for nucleotide sequences. Bioinformatics.

[40] Naidenov B, Lim A, Willyerd K, Torres NJ, Johnson WL (2019). Pan-genomic and polymorphic driven prediction of antibiotic resistance in *Elizabethkingia*. Front Microbiol.

[41] Edgar RC (2004). MUSCLE: a multiple sequence alignment method with reduced time and space complexity. BMC Bioinformatics.

[42] Kozlov AM, Darriba D, Flouri T, Morel B, Stamatakis A (2019). RAxML-NG: a fast, scalable and user-friendly tool for maximum likelihood phylogenetic inference. Bioinformatics.

[43] Bankevich A, Nurk S, Antipov D, Gurevich AA, Dvorkin M (2012). SPAdes: a new genome assembly algorithm and its applications to single-cell sequencing. J Comput Biol.

[44] Page AJ, Cummins CA, Hunt M, Wong VK, Reuter S (2015). Roary: rapid large-scale prokaryote pan genome analysis. Bioinformatics.

[45] Benjamini Y, Hochberg Y (1995). Controlling the false discovery rate: a practical and powerful approach to multiple testing. J R Stat Soc.

[46] Website https://www.health.gov.mw/index.php/health-ethics-standards?download=57:mstg-full-cover2015 (accessed 16 May 2020). https://www.health.gov.mw/index.php/health-ethics-standards?download=57:mstg-full-cover2015.

[47] Ministry of Health Malawi National Tuberculosis Control Programme Manual. https://pdf.usaid.gov/pdf_docs/PA00KGDD.pdf (accessed 15 May 2020). https://pdf.usaid.gov/pdf_docs/PA00KGDD.pdf.

[48] Feehily C, Karatzas KAG (2013). Role of glutamate metabolism in bacterial responses towards acid and other stresses. J Appl Microbiol.

[49] Afset JE, Bruant G, Brousseau R, Harel J, Anderssen E (2006). Identification of virulence genes linked with diarrhea due to atypical enteropathogenic *Escherichia coli* by DNA microarray analysis and PCR. J Clin Microbiol.

[50] Binns MM, Mayden J, Levine RP (1982). Further characterization of complement resistance conferred on *Escherichia coli* by the plasmid genes *traT* of R100 and *iss* of ColV,I-K94. Infect Immun.

[51] Binns MM, Davies DL, Hardy KG (1979). Cloned fragments of the plasmid ColV,I-K94 specifying virulence and serum resistance. Nature.

[52] Johnson TJ, Wannemuehler YM, Nolan LK (2008). Evolution of the *iss* gene in *Escherichia coli*. Appl Environ Microbiol.

[53] Tarr PI, Bilge SS, Vary JC, Jelacic S, Habeeb RL (2000). Iha: a novel *Escherichia coli* O157:H7 adherence-conferring molecule encoded on a recently acquired chromosomal island of conserved structure. Infect Immun.

[54] Guyer DM, Radulovic S, Jones F-E, Mobley HLT (2002). Sat, the secreted autotransporter toxin of uropathogenic *Escherichia coli*, is a vacuolating cytotoxin for bladder and kidney epithelial cells. Infect Immun.

[55] Nataro JP, Seriwatana J, Fasano A, Maneval DR, Guers LD (1995). Identification and cloning of a novel plasmid-encoded enterotoxin of enteroinvasive *Escherichia coli* and *Shigella* strains. Infect Immun.

[56] Mao B-H, Chang Y-F, Scaria J, Chang C-C, Chou L-W (2012). Identification of *Escherichia coli* genes associated with urinary tract infections. J Clin Microbiol.

[57] Tyler AD, Mataseje L, Urfano CJ, Schmidt L, Antonation KS (2018). Evaluation of Oxford Nanopore’s Minion sequencing device for microbial whole genome sequencing applications. Sci Rep.

[58] Bowden R, Davies RW, Heger A, Pagnamenta AT, de Cesare M (2019). Sequencing of human genomes with nanopore technology. Nat Commun.

