# Supplementary material for: Genomic epidemiology of Escherichia coli isolates from a tertiary referral center in Lilongwe, Malawi
Source: Microb Genom. 2020 Dec 9;7(1):mgen000490. doi: 10.1099/mgen.0.000490 (PMC8115906; doi:10.1099/mgen.0.000490)
Supplement: Supplementary material 1 [file mgen-7-490-s001.pdf]

SUPPLEMENTARY MATERIALS

Genomic Epidemiology of Escherichia coli Isolates from a Tertiary Referral Center in Lilongwe, Malawi

Gerald Tegha[1,*], Emily J. Ciccone[2,*], Robert Krysiak[1], James Kaphatika[1], Tarsizio Chikaonda[1], Isaac Ndhlovu[3], David van Duin[2], Irving Hoffman[2], Jonathan J. Juliano[2,4,5,+,&], Jeremy Wang[6,+]

Supplemental Methods

"Core" gene annotation and phylogeny reconstruction

We ran Prokka (v1.14.6) [cite https://pubmed.ncbi.nlm.nih.gov/24642063/] on each of our assembles genomes and the assemblies generated from the Illumina data from Musicha *et al*. [cite 27] with default parameters. We then ran Roary (v3.13.0) [cite https://academic.oup.com/mbe/article/37/3/933/5652084] with the following parameters: "--group-limit 1000000 -cd 90 -e --mafft" given the Prokka annotated genes (*.gff output) for our samples alone, and our samples plus the Musicha *et al*. samples (hereafter, the "joint" analysis). The "-cd 90" parameter sets the threshold or genes to be considered "core" to 90% of all samples. This is significantly relaxed from the default (99%) to increase the total number of genes with which we can build a reliable whole-genome phylogeny. For these two datasets, we ran RAxML-ng [cite 40] as described in the main methods (with "--model GTR+G") on the concatenated core gene alignments produce by Roary (core_gene_alignment.aln). For our data only, the core gene alignment included 791 genes, 387,856 sites, and 25,482 informative SNPs (ignoring gaps). For the joint data, the core alignment included 1,000 genes, 557,413 sites, and 60,972 informative SNPs.

In both the BUSCO and Roary-based phylogenetic analyses, we observe an apparent increase in substitution rate across the phylogeny when combining our data with the samples from Musicha *et al*. We suggest this difference could be a result of the temporal and geographical separation between the two datasets, i.e. although they share a similar distribution of ST clades, we expect the total within-study variation to be significantly lower than the between-study variation since they were individually sampled over a smaller temporal and geographical range. We also observe an increase in total substitution rate in the Roary trees relative to the BUSCO trees. We suggest this is a result of including a broader set of "core" genes than the initial set of BUSCOs (conserved single-copy orthologs) and are thus less-well conserved biologically.

Supplemental Figures

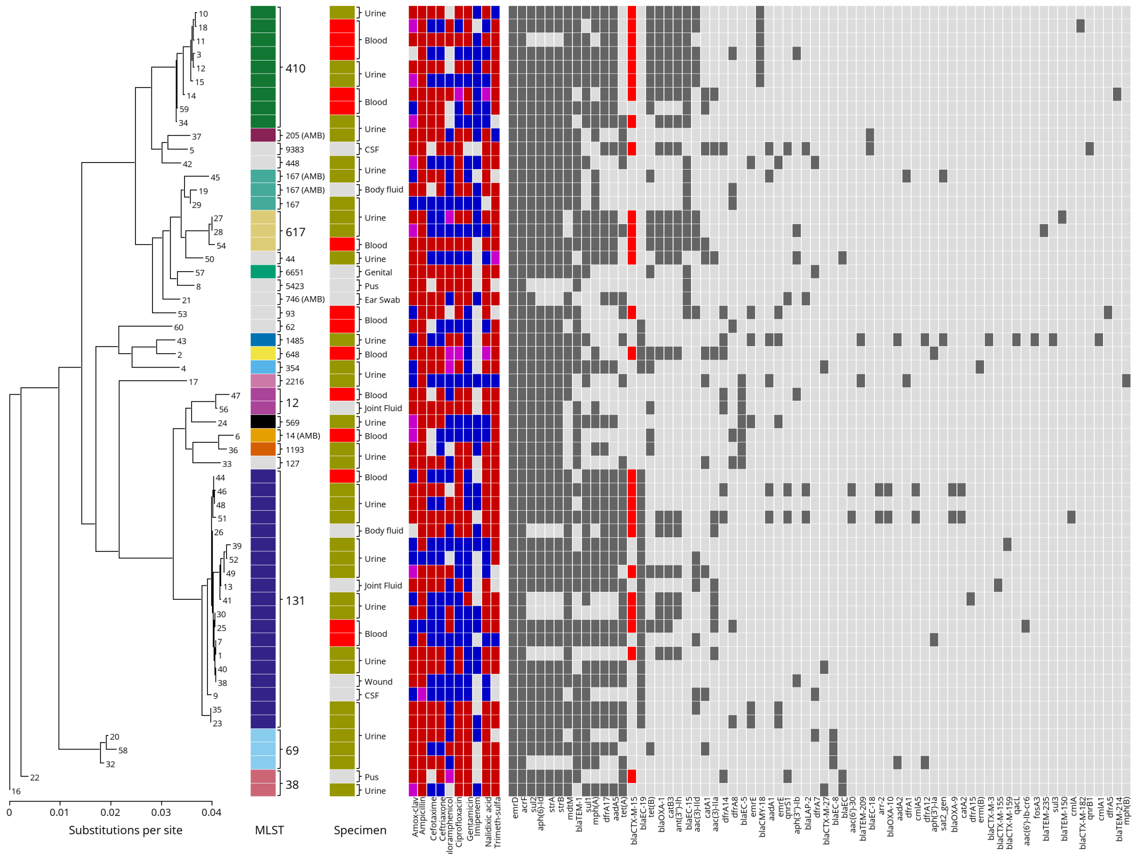

Supplemental Figure 1. Phylogenetic tree among 58 nanopore-based assemblies using Prokka gene annotations and Roary "core" gene alignment (with core genes present in >90% of samples). The alignment contains 791 genes, 387,856 sites, and 25,482 informative SNPs. The MLST, specimen type, and virulence/AMR factors are shown similar to Figure 1 in the main text. Despite using almost ten times as many genes as the BUSCO phylogenetic analysis, the major relationships within and among ST clades are largely preserved.

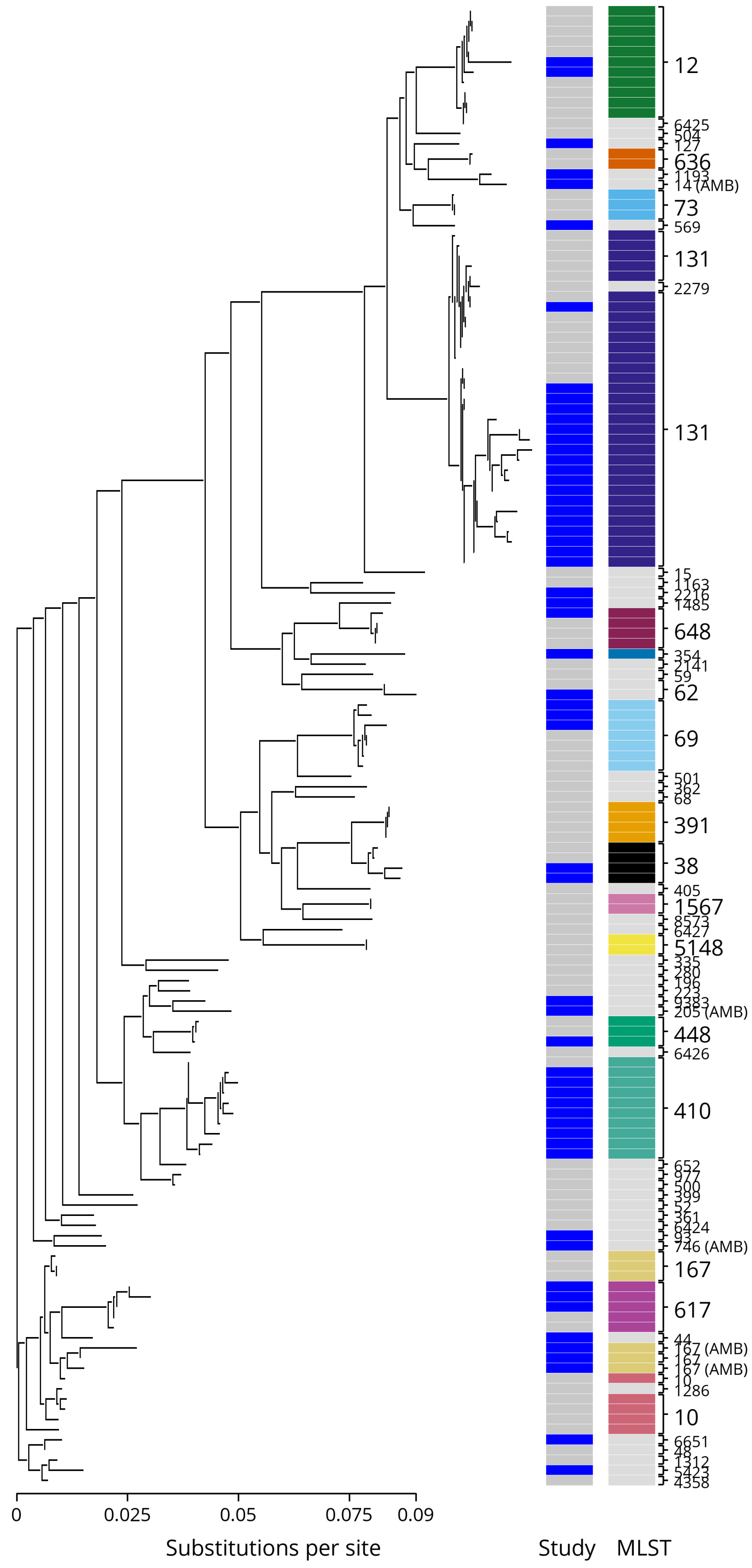

Supplemental Figure 2. Phylogenetic tree among 146 nanopore-based assemblies and Illumina assemblies from Musicha *et al*. using Prokka gene annotations and Roary "core" gene alignment (with core genes present in >90% of samples). The alignment contains 1,000 genes, 557,413 sites, and 60,972 informative SNPs. Despite using over ten times as many genes as the BUSCO phylogenetic analysis presented in Figure 2 (main text), the major relationships within and among ST clades are largely preserved, including paraphyletic relationships observed among STs 131 and 2279, STs 12 and 6425, and STs 10, 167, and 617.

Supplemental Tables

Supplemental Table 1. Databases Used for Sequence Analysis

Database	Version/Date	Ref
MLST	https://bitbucket.org/genomicepidemiology/mlst_db/commits/94abfd0 (2020-03-10)	Center for Genomic Epidemiology
pMLST	https://bitbucket.org/genomicepidemiology/pmlst_db/commits/a100502 (2020-03-01)	Center for Genomic Epidemiology
VirulenceFinder	https://bitbucket.org/genomicepidemiology/virulencefinder_db/commits/13d72a8 (2020-03-01)	Center for Genomic Epidemiology
fimTyper (fimH)	https://bitbucket.org/genomicepidemiology/fimtyper_db/commits/f999a42 (2020-03-10)	Center for Genomic Epidemiology
SRST2 (serotype)	https://github.com/katholt/srst2/commit/fe027e55 (2020-03-01)	http://genomemedicine.com/content/6/11/90
AMRFinderPlus	3.6 (2020-01-06.1)	NCBI AMR Database (https://www.ncbi.nlm.nih.gov/bioproject/PRJNA313047)

Supplemental Table 2: Public Sequences Used in Analysis

Source	Strain ID	ENA Accession ID
Musicha et al.	A39011	ERS668966
Musicha et al.	522_A	ERS668975
Musicha et al.	A45214	ERS668976
Musicha et al.	1010805	ERS668977
Musicha et al.	BHA15G	ERS668978
Musicha et al.	BKQ7M8	ERS668979
Musicha et al.	BKR1Z7	ERS668980
Musicha et al.	D40034	ERS668981
Musicha et al.	BKQ5JN	ERS668982
Musicha et al.	A7898	ERS668983
Musicha et al.	3361	ERS668984
Musicha et al.	D3787	ERS668967
Musicha et al.	BKR406	ERS668987
Musicha et al.	D3475	ERS668988
Musicha et al.	B12381	ERS668989
Musicha et al.	A7503	ERS668990
Musicha et al.	C301	ERS668991
Musicha et al.	A38084	ERS668992
Musicha et al.	D3275	ERS668993
Musicha et al.	A5175	ERS668994
Musicha et al.	B1PG3	ERS668968
Musicha et al.	D4531	ERS668995
Musicha et al.	2473	ERS668996
Musicha et al.	D3420	ERS668998
Musicha et al.	C10382	ERS668999
Musicha et al.	2228	ERS669000
Musicha et al.	10151	ERS669001
Musicha et al.	8728	ERS669002

Musicha et al.	10129	ERS669004
Musicha et al.	A36329	ERS669005
Musicha et al.	C1289	ERS668969
Musicha et al.	B9070	ERS669006
Musicha et al.	9597	ERS669007
Musicha et al.	D36115	ERS669008
Musicha et al.	2209	ERS669009
Musicha et al.	A25576	ERS669010
Musicha et al.	D37334	ERS669011
Musicha et al.	D25640	ERS669012
Musicha et al.	D25641	ERS669013
Musicha et al.	D29454	ERS669014
Musicha et al.	A38988	ERS669015
Musicha et al.	1012184	ERS668970
Musicha et al.	A40286	ERS669016
Musicha et al.	4464	ERS669017
Musicha et al.	B9222	ERS669018
Musicha et al.	C14036	ERS669019
Musicha et al.	A36140	ERS669020
Musicha et al.	3524	ERS669021
Musicha et al.	4600	ERS669022
Musicha et al.	C12359	ERS669024
Musicha et al.	A32883	ERS669025
Musicha et al.	BKQA8N	ERS668971
Musicha et al.	9693	ERS669027
Musicha et al.	2558	ERS669028
Musicha et al.	A27	ERS669029
Musicha et al.	C29	ERS669031
Musicha et al.	A333	ERS669032
Musicha et al.	D39719	ERS669033

Musicha et al.	C30	ERS669034
Musicha et al.	C15	ERS669035
Musicha et al.	D40059A	ERS669036
Musicha et al.	BHAIAI	ERS668972
Musicha et al.	D43713	ERS669037
Musicha et al.	C4	ERS669039
Musicha et al.	A16	ERS669041
Musicha et al.	A35440	ERS669042
Musicha et al.	C33B	ERS669044
Musicha et al.	D32322	ERS669045
Musicha et al.	C14	ERS669047
Musicha et al.	D3871	ERS669048
Musicha et al.	1014142	ERS669049
Musicha et al.	D29253	ERS668973
Musicha et al.	B28	ERS669050
Musicha et al.	C20b	ERS669051
Musicha et al.	D49086	ERS669052
Musicha et al.	D48799	ERS669054
Musicha et al.	B3	ERS669055
Musicha et al.	1016948	ERS668974
Musicha et al.	BKQ79K_1	ERS669067
Musicha et al.	10276	ERS669087
Musicha et al.	D26076	ERS669089
Musicha et al.	A7881	ERS669101
Musicha et al.	D45621	ERS669107
Musicha et al.	D4275	ERS669114
Musicha et al.	A44893	ERS669128
Musicha et al.	A1a	ERS669146
Musicha et al.	10140	ERS668997
Musicha et al.	D42544	ERS669030

Musicha et al.	D46760	ERS669038
Musicha et al.	A48349	ERS669040
Musicha et al.	A45016	ERS669046
Musicha et al.	A3b	ERS669056
Musicha et al.	D33237	ERS669057

Supplemental Table 3: Distribution of Sequence Types (ST) in 58 Included Isolates

Sequence Type (ST)	Number	Frequency
131	19	32.8%
410	9	15.5%
69	3	5.2%
38	2	3.4%
617	3	5.2%
12	2	3.4%
354	1	1.7%
5423	1	1.7%
2216	1	1.7%
569	1	1.7%
127	1	1.7%
1193	1	1.7%
58	1	1.7%
62	1	1.7%
93	1	1.7%
167	1	1.7%
9385	1	1.7%
1485	1	1.7%
648	1	1.7%
44	1	1.7%
6651	1	1.7%
14/18/1416/1540/1666/1927/6460/9139/9779	1	1.7%
167/2815/2821/4183/5507	1	1.7%
167/693/694/1417/2266/2504/3015/4189/4815/5018/6892/7611/9622	1	1.7%
205/341/2539/5296/5960/7303/7955	1	1.7%
746/1144/2601/6225/6581/7178/8221/9447	1	1.7%

Supplemental Table 4: Detected O Groups in 58 Included Isolates

O Antigen	Count	Frequency
O25	15	25.9%
O8	6	10.3%
Onovel32	4	6.9%
O153var1	4	6.9%
ND	3	5.2%
O18	2	3.4%
Onovel14	2	3.4%
O9	2	3.4%
O16	2	3.4%
O4	2	3.4%
O7	2	3.4%
O84	1	1.7%
O11	1	1.7%
O86	1	1.7%
O24	1	1.7%
O15	1	1.7%
O134	1	1.7%
O45	1	1.7%
O6	1	1.7%
Onovel1	1	1.7%
O75	1	1.7%
O100	1	1.7%
O29	1	1.7%
O17	1	1.7%
Onovel32/O9	1	1.7%

ND: Not Determined

Supplemental Table 5. Detected H Groups in 58 Included Isolates

H-Type	Number	Frequency
H4	20	34.5%
H9	12	20.7%
H10	4	6.9%
H18	3	5.2%
H5	5	8.6%
H21	2	3.4%
H31	2	3.4%
H1	1	1.7%
H6	1	1.7%
H12	1	1.7%
H16	1	1.7%
H23	1	1.7%
H30	1	1.7%
H34	1	1.7%
H37	1	1.7%
H42	1	1.7%
H45	1	1.7%
